# The Challenge of maintaining good health outcomes for all during the COVID‐19 pandemic—a view from family medicine in Wales, UK

**DOI:** 10.1002/jgf2.363

**Published:** 2020-11-01

**Authors:** Sally Lewis

**Affiliations:** ^1^ Welsh Government Department for Health Social Services and Children Cardiff UK; ^2^ Swansea University School of Medicine Swansea UK

## Abstract

This article addresses the issue of maintaining essential healthcare services throughout the pandemic and beyond. It suggests a key role for the use of patient‐reported outcomes.

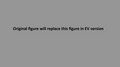

The COVID‐19 pandemic has generated an extraordinary response from the healthcare system in transforming the way that we work in order that we operate in a way that minimizes spread of infection and allows services to cope during a surge of cases of viral infection. However, this came at a price with the temporary cessation of virtually all outpatient and elective activity in hospitals. We have in our response moved to remote triage for all patients in primary care and have seen a rapid rise in the use of video consulting to support safe decision making. Face‐to‐face consultations have been minimized, and QOF activity has been formally postponed.

Change has happened at great speed and been made possible through a considerable cutting of red tape, using the goodwill and flexibility of the workforce and digitally supported remote management. Some of this system change in healthcare delivery is positive and should be captured and adopted for the longer‐term benefit of everyone. We have a duty to ensure that services essential for people with non‐COVID time‐sensitive problems are able to function so that outcomes are not adversely affected by the necessary pandemic response.

The importance of this is heightened as we know that it is likely that society will be living with this pandemic until a vaccination is available. For this reason, continued vigilance and flexibility are needed to be able to step up the specific responses to the virus as necessary.

This is a highly complex and uncertain scenario which requires decisions to be made within an ethical framework in order that we balance the competing risks to achieve the best outcomes for the whole population. A focus on equity is needed, equity in geographical provision of services and also between patient groups with differing needs. Particular attention is required to work against inequities caused by social deprivation and ethnicity which may grow during the pandemic, as well as vulnerable groups such as those with learning disabilities, care home residents, or those needing palliative care.

There will be competing demands from different specialties on supporting services such as radiology, pathology, and theaters as colleagues begin to tackle the backlog of people waiting for treatment. Undoubtedly, there will be a need for prioritization of those in greatest need to be brought to the front of the queue. In Wales, we try to be governed by the principles of prudent and value‐based health care. One of these principles is to “Care for those with the greatest health need first” and is therefore concerned with equity in healthcare provision. This should be how we are guided as we switch services back on, particularly as capacity will inevitably be reduced because of stringent infection control measures.

It will not be easy at all as we put the wheels back on the wagon, still with the threat of COVID‐19 hanging over us, the will to deliver against a plethora of gold standard single disease guidelines and the ongoing need to avoid healthcare‐transmitted viral infection. A focus on outcomes across the board is therefore essential for maintaining equitable health care during the pandemic.

Lockdown is not benign, neither is the virus. We must ensure that essential services for life‐threatening, life‐changing, or time‐sensitive conditions are maintained and that outcomes for those with non‐COVID illness do not suffer. Achieving equitable outcomes with the resources available to us is how we understand value in health care. The only way to know whether we are achieving this, and to avoid otherwise invisible harm, is to track outcomes. It is insufficient to measure access to services alone.

We must use all tools available to us to safely meet the needs of greater numbers of people in ways that do not always require a physical visit to a healthcare facility. Remote management is necessary to protect patients from exposure to infection during the pandemic but is also arguably often less of a burden in terms of travel for those who prefer it. Telephone and video consultations do not increase capacity in the system as they take up clinician time, but “asynchronous” communication can be a tremendous aid to sustainability. Patient‐reported outcomes allow structured communication, enabling triage and informing flexible and patient‐led access to their clinical teams.

Of particular note so far has been the growth in enthusiasm for rolling out patient‐facing technology. That has really helped us to take a leap forward with the digital communication necessary for us to capture PROMs at scale and in a way that is meaningful and useful for patients.

Because of the need to manage more vulnerable groups remotely, there has also been a renewed interest in how we tackle issues of digital exclusion. Large numbers of people were “shielded” and have to be managed remotely. PROMs offer a structured assessment to support safe remote management as part of a package of care. They will also be critical in how we manage the back log of patients waiting for treatment in the post‐COVID recovery phase to help us prioritize safely and meet the greatest need first.

In many ways, the pandemic has created the perfect storm for the NHS and we will not have the luxury of returning to how things were if we are to treat all our patients, and equitably maintain outcomes. It is more important than ever to focus on value for all patients and so the post‐COVID recovery phase should be characterized by a process of stop, resume, and reset:

Stop—there are undoubtedly some low‐value practices (from everybody’s perspective) that we should stop for good. I am thinking about unnecessary imaging, procedures of limited evidence, and appointments which leave patients wondering why they made the trip.

Resume—there are things that we need to resume quickly to maintain good outcomes. This cannot be a bun‐fight of the specialities. We must work collectively if we are to ensure the best outcomes, prioritizing the time‐sensitive issues.

Finally, we should be brave enough to reset through capitalizing on learning from the pandemic to deliver higher value care for our patients in the future in ways that are sustainable and meet our needs in the 21st century.

## CONFLICT OF INTEREST

The authors have stated explicitly that there are no conflicts of interest in connection with this article.

